# Stability of corneal endothelial monolayers in the presence of magnetic particles in a rotating magnetic field

**DOI:** 10.1371/journal.pone.0348603

**Published:** 2026-05-06

**Authors:** Jessica Chin, Leah A. Marquez-Curtis, Mehdi Ghaffari Sharaf, Janet A. W. Elliott, Larry D. Unsworth

**Affiliations:** 1 Department of Biomedical Engineering, University of Alberta, Edmonton, Alberta, Canada; 2 Department of Chemical and Materials Engineering, University of Alberta, Edmonton, Alberta, Canada; 3 Department of Laboratory Medicine and Pathology, University of Alberta, Edmonton, Alberta, Canada; Children’s Hospital of Orange County, UNITED STATES OF AMERICA

## Abstract

Magnetic particles have significant potential as novel theranostics due to the relative ease of surface modification for targeting molecules or regions of diseased tissues, coupled with remote *in vivo* manipulation using an exogenous magnetic field. Employing magnetic particles within the eye is hindered by the lack of safety studies. In particular, our lab has developed peptide-conjugated iron-oxide magnetic particles that target and liberate pseudoexfoliation materials from anterior lens capsules under a rotating magnetic field. Further application of such a mode of therapy may hold the key to reducing the incidence of open-angle glaucoma but requires an evaluation of its potential cytotoxic effects on delicate ocular tissue like the cornea, where a disruption of the endothelial cells can cause blindness. To this end, the effects of peptide-conjugated magnetic particles in a low-frequency rotating magnetic field on cultured porcine corneal endothelial cell monolayers was characterized using membrane integrity assessments for cell viability, metabolic activity assay, and ZO-1 immunocytochemical staining. It was observed that magnetic particles under the influence of this magnetic field had no significant immediate or delayed effect on membrane integrity or metabolic activity of corneal endothelial cell monolayers in culture. Moreover, the expression of the ZO-1 tight junction protein was maintained in the monolayers following magnetic treatment. All results support the safe application of 1-µm magnetic particles (0.2 µg/µL) and a rotating magnetic field (5.5 Hz and 0.5 T) for up to 3 hours as a novel therapy for pseudoexfoliation glaucoma.

## Introduction

Eye diseases are often challenging to treat due to the delicate design and sophisticated function of ocular structures. A promising tool that could greatly increase the possible treatment options within the eye are iron oxide magnetic particles (MPs) under external magnetic fields. These micro-/nanosized particles are small enough to interact at the cellular and molecular scales [1–3] allowing for the introduction and movement of these particles within the ocular environment. Iron oxide is the most commonly used material for MPs designed for biomedical purposes due to its biocompatibility, stability, and magnetic properties that allow MPs to be mechanically actuated within physiological conditions upon the application of an external magnetic field [1,3–5]. Iron oxide MPs are also biodegradable, metabolized by lysosomes in cells [6,7], removing any risks of MP accumulation in the eye. These particles are often designed with targeting ligands, allowing for the particles to find and bind to specific biomolecules or diseased tissues [2,8,9]. The ability to remotely direct the movement of MPs within the eye to target structures has garnered interest and research into the use of these magnetic systems for drug [10–12] and cell delivery [13–15] applications. Apart from eye treatments, these mechanically actuated MPs are often utilized for the destruction of malignant cells by inducing cell apoptosis pathways through mechanical triggers [16,17], or the direct rupture of cell [18] or lysosome [19] membranes. Our group has also developed pseudoexfoliation-targeting MPs for the targeted removal of pseudoexfoliation materials from anterior lens capsules *ex vivo* under an external low-frequency rotating magnetic field [20]. The most likely future clinical administration of MPs is to the anterior segment region, examples of which have been studied for other therapeutic particles [20–23]. The effect of the mechanical motion of these particle types on corneal endothelial cells under a magnetic field remains understudied but is crucial for advancing these therapeutic interventions. The corneal endothelial monolayer plays a key role in maintaining the transparency of the cornea [24], and therefore clear vision. However, any damage to this delicate layer can result in irreversible corneal blindness due to the limited regenerative ability of corneal endothelial cells *in vivo* [25,26]. It is therefore important to uncover any potential negative effects resulting from the application of MPs under magnetic fields on corneal endothelial cells.

Several groups have applied MPs under an exogenous magnetic field in the anterior chamber [27–29]. An *in vitro* study tested 50-nm MPs engulfed by human corneal endothelial cells and concentrated the cells on contact lenses using permanent magnets (up to 0.16 T, theoretical maximum of 1.46 T) [28]. The strongest field strength of 0.16 T resulted in the most significant increase in cell density compared to untreated cells. No decrease in cell viability was observed for cells that endocytosed the 50-nm MPs, and Zonula Occludens (ZO-1) tight junctions were observed in the magnetic human corneal endothelial cells at 1 and 5 days *in vitro*. In another study, human corneal endothelial cells treated with 50-nm MPs were applied to Descemet’s membrane-stripped rabbit eyes and a cylindrical neodymium magnet (12 mm D x 20 mm H) was taped to the closed eyelid over the cornea for 3 hours [29]. Monolayer formation of the injected cells was confirmed through the presence of ZO-1, neural cell adhesion molecule, N-Cadherin, and CD166 markers in cryo-sectioned corneas. Increased corneal clarity of the rabbit eyes was recorded 3 and 7 days after treatment [29]. Similar techniques have been used to transplant cultured cells to create a viable and functional monolayer 12 months after injecting magnetic rabbit corneal endothelial cells [23]. In all cases, no decrease in cell viability following the addition and subsequent endocytosis of the MPs in corneal endothelial cell cultures was observed, suggesting the safe use of MPs and magnetic fields *in vitro* and *in vivo* in the eye. However, these studies were interested in using magnetic properties to manipulate the location and attachment of cells to existing regions of a monolayer. In contrast, our previous study exploited the potential use of MPs in a dynamic rotating magnetic field to degrade pseudoexfoliation materials [20]. It remains unclear if low-frequency magnetic field oscillation (1–4 Hz) of MPs will be cytotoxic to corneal endothelial cells. For example, low-frequency oscillating magnetic fields did not induce a decrease in neuron cell viability in the presence of MPs (1–3 Hz, 320 mT) [30]. A later study using a similar magnetic field (1–4 Hz, 450 mT) showed that low-frequency oscillating fields could be cytotoxic to cancer cells [31]. The observation that similar treatment conditions can lead to different viability outcomes motivates the testing for any negative effects that may result from the application of MPs in a low-frequency rotating magnetic field. This is particularly important for corneal endothelial cell monolayers despite previous biosafety studies in rabbit models showing that MPs in an exogenous magnetic field may be safe for application in the cornea [27,29].

In this work, the cytotoxic effects of MPs in a rotating magnetic field on porcine corneal endothelial cell (PCEC) monolayers were examined. PCECs were used because they resemble human corneal endothelial cells in shape and function [32], thus providing an accessible model for the human corneal endothelium; a requirement due to the high demand for human corneas for patient transplantation [33]. PCEC monolayer cultures were exposed to 0.2 µg/µL of MPs and a 5.5 Hz, 0.5 T rotating magnetic field, following similar conditions previously used for MPs in the context of therapeutic applications [20], as well as in oscillating field studies that used similar frequencies [31]. The magnetic field was applied for 3 hours because it represents an extreme upper time limit for any clinical visit while allowing for evaluation of the system, and some studies have shown some therapies (e.g., pseudoexfoliation therapies) may require this length of time [20]. Cell membrane integrity (as a surrogate measure of cell viability), AlamarBlue metabolic activity, and immunocytochemical expression of the tight-junction protein ZO-1 were assessed to determine cell monolayer damage due to the magnetic treatment. Assessments were performed immediately following magnetic treatment and after an overnight incubation period post-treatment to assess any immediate or delayed injury to the cells. Understanding any effects that these MPs and a rotating magnetic field have on the corneal endothelial monolayer will inform their future use in the anterior chamber of the eye.

## Methods

### Peptide conjugation to MPs

Pseudoexfoliation-targeting MPs were generated using previously published procedures [20]. Briefly, 1-µm iron oxide polymer-coated MPs with azide surface functionalization (Nanocs Inc., New York, USA) were conjugated with alkyne-modified synthetic peptides with the pseudoexfoliation-targeting sequences IPLLNPGSMQLS (p-IPL) and LPSYNLHPHVPP (p-LPS) (RS Synthesis, Louisville, KY, USA). Copper catalyzed azide-alkyne click chemistry was used to conjugate the peptides to the MPs as described previously [20]. Characterization of the magnetic particle-peptide conjugates (MP-p-IPL and MP-p-LPS) was done using zeta potential measurements, Fourier-transformed infrared spectroscopy (FTIR), and a competitive inhibition assay [20].

### Cell cultures

PCECs were isolated from pig eyeballs as previously described [34]. The use of primary porcine cells was exempted for review by the Animal Care and Use Committee of the University of Alberta because the eyes were obtained from pigs slaughtered for meat consumption and not for the sole purpose of this research. Briefly, the corneoscleral tissue was excised and the endothelial layer was treated with trypsin/EDTA (0.05%/0.02%, Gibco, Life Technologies, Grand Island, NY, USA). The enzymatically-dislodged cells were re-suspended in DMEM supplemented with 4.5 mg/mL glucose, 4 mM L-glutamine, 10% fetal bovine serum, and 1% antibiotic–antimycotic agent (10,000 units/mL of penicillin, 10,000 µg/mL of streptomycin, and 25 µg/mL of amphotericin B), hereinafter referred to as PCEC medium (all components were from Gibco). The cells were seeded in T25 tissue culture flasks (430639, Corning Inc., NY, USA) and incubated at 37 °C and 5% CO_2_ until confluent. Media changes were carried out every 2–3 days. Once confluent, the primary cells were trypsinized (TrypLE Express, Gibco) and counted using a Coulter® Z2^TM^ particle count and size analyzer (Beckman Coulter, Mississauga, ON, Canada), then sub-cultured or cryopreserved for future experiments.

It was previously shown that PCECs can retain high viability following optimal cryopreservation [34,35]. For these experiments, we used PCECs that were cryopreserved in the following manner: one mL cell suspensions were equilibrated on ice in PCEC medium containing 5% (w/w) dimethyl sulfoxide (DMSO, D-128, Fisher Scientific, Fair Lawn, NJ, USA) and 6% (w/w) hydroxyethyl starch (PST002, Preservation Solutions, Inc., Elkhorn, WI, USA) in plastic cryovials (Nunc 1.8 mL CryoTube vials, ThermoFisher Scientific, Waltham, MA, USA) for 15 minutes. The cryovials were then placed in a CoolCell container (Corning), and kept in a –80 °C freezer (providing a cooling rate of approximately 1 °C/minute). The next day the cryovials were transferred to liquid nitrogen for long-term storage. The cells were rapidly thawed in a 37 °C water bath (VWR, Mississauga, ON, Canada), resuspended in PCEC medium, and seeded in culture flasks or glass coverslips in a 24-well culture plate as described below.

Glass coverslips (clear borosilicate glass, 72190–09, 12 mm, Electron Microscopy Sciences, Hatfield, PA, USA) were autoclaved and further sterilized by immersing in 70% isopropanol (LC157602, LabChem Inc., Zelienople, PA, USA) in a sterile Petri dish (100 x 15 mm, Fisher Scientific) for at least 30 minutes, then transferred to phosphate-buffered saline (1X PBS, 100-10-023, Life Technologies) for 15 minutes before placing into a 24-well plate (Cellstar®, 662160, Greiner Bio-One, Monroe, NC, USA). Fibronectin solution was prepared by adding 18 µL of fibronectin from stock solution (1 mg/mL, F-1141, Sigma-Aldrich, Oakville, ON, Canada) to 982 µL of 1X PBS resulting in a fibronectin concentration of 18 μg/mL. To coat coverslips with fibronectin to promote attachment of PCECs, first, excess PBS on coverslips was removed gently by pipetting. Then 100 µL of fibronectin solution was added to each coverslip ensuring coverage of the entire area, incubated for at least 30 minutes at room temperature (21 °C), and then removed. The PCECs (passage 2–4) were seeded at a density of 40,000–60,000 cells/cm^2^ on fibronectin-coated glass coverslips in 500 µL of PCEC medium and allowed to grow to 80–100% confluence, with medium changes every 2–3 days.

### Description of control and experimental conditions

Positive controls (live cells) consisted of five coverslips with untreated confluent PCEC monolayers. Negative controls (dead cells) consisted of three coverslips with confluent PCEC monolayers, plunged directly into liquid nitrogen, kept there for 1 hour, then rapidly thawed in a 37 °C water bath. The coverslips were stained with SYTO 13/GelRed and assessed for cell viability as described below. Because the experiments were to be carried out with live cell monolayers at room temperature (i.e., 21 °C) outside the incubator, we also examined whether cell viability would be affected when the PCEC monolayers were left outside the incubator for the duration of the experiments. Three coverslips each with untreated confluent PCEC monolayers were assessed for cell viability after exposing them to room temperature conditions for 30 minutes and 3 hours.

For magnetic field experiments, three coverslips each with confluent PCEC monolayers were treated with virgin (bare MPs) or peptide-modified MPs (MP-p-IPL or MP-p-LPS) in 50 µL water, resuspended in 450 µL medium (final concentration: 0.2 µg/µL), exposed to a rotating magnetic field (5.5 Hz, 0.5 T bar magnet) for 3 hours at room temperature, then assessed for cell viability. As a control for magnetic field application without MPs, PCEC monolayers in four coverslips each in cell medium only (500 µL) were exposed to a rotating magnetic field for 3 hours (5.5 Hz, 0.5 T bar magnet), then assessed for cell viability. The total numbers of cells in these control coverslips were used in the calculation of absolute viability as described below.

To exclude the effect of delayed injury on the cells [35] following the exposure to MPs under a magnetic field, confluent PCEC monolayers were assessed immediately after treatment and also after overnight incubation (18–20 hours) post-treatment. PCEC monolayers on coverslips were treated with cell medium only (500 µL), or with MPs in 50 µL water, resuspended in 450 µL medium (final concentration: 0.2 µg/µL), namely bare MPs, MP-p-IPL, and MP-p-LPS, and exposed to magnetic field for 3 hours at room temperature. Three coverslips for each experimental condition were assessed immediately for cell viability using SYTO 13/GelRed. For another three coverslips for each experimental condition, the medium containing the MPs was pipetted out from each well after exposure to magnetic field, and replaced with 500 µL of fresh medium. The coverslips were then placed in a 37 °C and 5% CO_2_ incubator overnight (18–20 hours) and assessed for cell viability using SYTO 13/GelRed.

### Cell viability assessment using fluorescence microscopy

The cell viability was assessed based on membrane integrity using SYTO 13 and GelRed dyes. SYTO 13 permeates all cells (both membrane-intact and membrane-damaged) and complexes with nucleic acids to emit a green fluorescence. On the other hand, GelRed, a stronger nucleic acid binding dye that permeates only cells with damaged or compromised membranes, and had been validated as an alternative to ethidium bromide and propidium iodide [36,37], emits an intense red fluorescence which masks the SYTO 13 green fluorescence. Therefore membrane-intact (live) cells fluoresce green, while membrane-damaged (dead) cells fluoresce red. To prepare the SYTO 13/GelRed solution, 10 µL of GelRed (stock solution: 10,000X in water, 41003, Biotium, Scarborough, ON, Canada) and 10 µL of SYTO 13 (5 mM solution in DMSO, S7575, Molecular Probes, Eugene, OR, USA) were added to 262.5 µL of 1X PBS in a microtube (final concentrations: 354X GelRed and 0.18 mM SYTO 13) and kept on ice in the dark. Cell monolayers on glass coverslips in 24-well plates were stained by adding 15 µL of SYTO 13/GelRed solution to each well containing 285 µL of 1X PBS; the coverslips were then incubated at room temperature for 5 minutes in the dark. Each coverslip was then transferred (cell-side down) onto a glass slide (12–500-A3, Fisher Scientific), and observed under a fluorescent microscope (Dialux 22, Leitz, Germany) at 100X magnification. At least 5 images were captured per coverslip using an Infinity3 camera and Infinity Capture software (Lumenera Corporation, Ottawa, ON, Canada). Automated cell counting was carried out using the Viability3 program (custom software by Locksley McGann, Version 3.2, The Great Canadian Computer Company, Spruce Grove, AB, Canada), which outputs data on the total number of cells, and the numbers of green-fluorescing and red-fluorescing cells. The percentage relative membrane integrity or relative viability was calculated as:


$$\text{Relative viability }(\%)=\frac{\text{number of green cells on a coverslip }}{\text{total number of cells on a coverslip}}\times \text{100}\%$$ (1)

The % relative viability does not account for cell detachment; therefore, it can give an overestimated value when the average total cell count on the coverslip is decreased due to cells that get detached. To account for this, the % absolute viability is calculated follows:


$$\text{Absolute viability }(\%)=\frac{\text{number of green cells on an experimental coverslip}}{\text{total cell count on a medium only control coverslip}}\times \text{100}\%$$ (2)

### Metabolic activity assessment

To determine whether membrane-intact viable cells are also metabolically active, we employed the AlamarBlue reduction assay. AlamarBlue contains the cell-permeable and non-toxic indicator dye resazurin, which is blue in its oxidized form. Metabolically active cells are able to convert resazurin to resorufin, which is red in its reduced form. The colorimetric signal detected at 570 nm is proportional to the number of cells with innate metabolic activity in the sample. Because there is considerable overlap in the absorption spectra of the oxidized and reduced forms of AlamarBlue, absorbance was measured at two wavelengths namely 570 nm and 600 nm (SpectraMax Plus, Molecular Devices, San Jose, CA, USA).

For these experiments, PCECs were seeded in 96-well plates (13,000 cells/well in 200 µL of medium), and cultured to confluency. Six wells were used for each experimental treatment, namely cell medium only, MP-p-IPL, and MP-p-LPS, and exposed to magnetic field for 3 hours at room temperature. Three wells of PCEC monolayers for each experimental condition were assessed immediately for metabolic activity using AlamarBlue. For the other set of three wells for each experimental condition, the medium containing the MPs was pipetted out from each well and replaced with 200 µL of fresh medium. The plate was incubated in a 37 °C and 5% CO_2_ incubator overnight. The next day, the medium was replaced with 200 µL of fresh medium. For both the immediate and overnight assessments, 20 µL of AlamarBlue reagent (Invitrogen, Life Technologies) was added to 200 µL of medium covering the monolayers, and to wells containing medium only and no cells, and the plate incubated for 4 hours at 37 °C. After the incubation period, 100 µL of the medium from each well was transferred to a fresh 96-well plate. Absorbance was measured at 570 nm and 600 nm and the percent reduction of AlamarBlue was calculated as per manufacturer’s instructions with corrections applied for no-cell controls as previously described [38].

### Immunocytochemistry for ZO-1

Immunocytochemical staining of the tight junction protein ZO-1 in confluent PCEC monolayers on coverslips was also carried out immediately and after overnight incubation following magnetic field exposure. Two coverslips for each experimental condition were treated with medium only (500 µL), or with MPs in 50 µL water resuspended in 450 µL medium (final concentration: 0.2 µg/µL), namely bare MPs, MP-p-IPL, and MP-p-LPS, and exposed to magnetic field for 3 hours at room temperature. One coverslip for each experimental condition was assessed immediately for ZO-1 expression. For the other coverslip, the medium containing the MPs was pipetted out from each well and replaced with 500 µL of fresh medium. The coverslips were then placed in a 37 °C and 5% CO_2_ incubator overnight and assessed for ZO-1 expression the next day. The medium was removed and the cell monolayers washed with 500 µL of 1X PBS. The cells were fixed with 300 µL/well of 4% paraformaldehyde (Sigma) in 1X PBS for 15 minutes at room temperature, followed by washing with 500 µL of 1X PBS three times. Permeabilization of paraformaldehyde-fixed cells was performed using 0.1% Triton X-100 (Fisher Scientific) in 1X PBS for 15 minutes at room temperature followed by blocking with 3% bovine serum albumin (Sigma) in 1X PBS for at least an hour in the dark at room temperature. Fluorochrome-conjugated ZO-1 antibody (monoclonal antibody Alexa Fluor 488, 339188, Invitrogen, ThermoFisher Scientific; stock concentration: 0.5 mg/mL) was diluted in 1X PBS containing 3% bovine serum albumin and 0.3% Triton X-100 (1:100 dilution), and incubated with the cell monolayer overnight at 4 °C. The antibody was removed, and the monolayer washed three times with 1X PBS. The coverslips were mounted on glass slides and imaged using a fluorescent microscope (Leitz, Dialux 22) at 250X magnification.

### Statistical analysis

Either two-sample t-test or one-way ANOVA was performed using Origin Version 2024 (OriginLab Corporation, Northampton, MA, USA) to identify statistical significance. For all tests, a p-value < 0.05 was considered to be significant.

## Results

### Membrane integrity assessment of cell viability of controls

**Fig 1:**
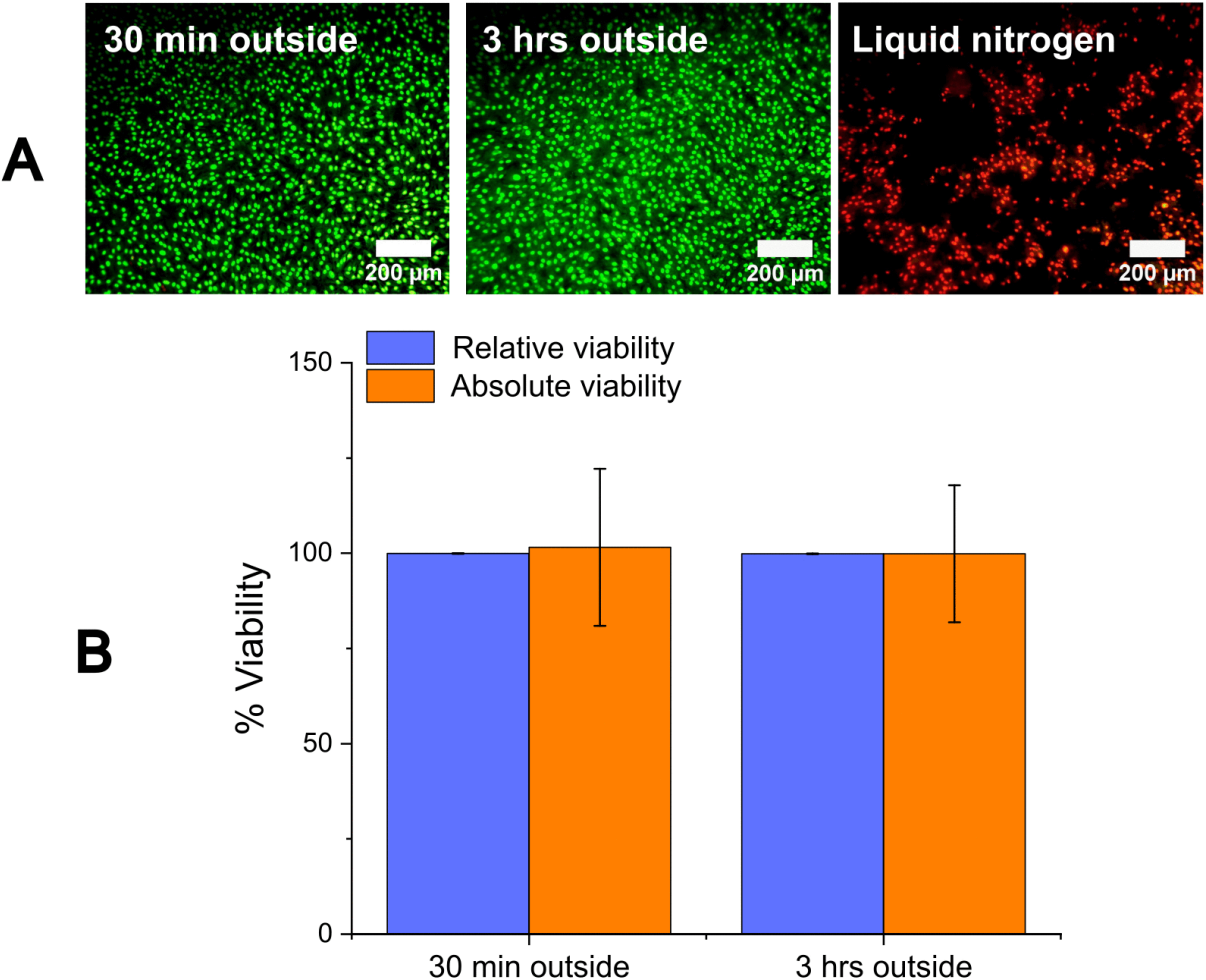
Viability assessment by fluorescent imaging of membrane integrity. Porcine corneal endothelial cell monolayers were left outside of temperature- and CO_2_-controlled incubator cell culture conditions for 30 minutes and 3 hours. **(A)** Representative images of porcine corneal endothelial cell monolayers stained with SYTO 13/GelRed (100X magnification). Green fluorescence indicates intact cell membranes while red fluorescence indicates damaged cell membranes. No viable cells are seen after a plunge into liquid nitrogen. **(B)** Percentage relative viability (blue bars) and absolute viability (orange bars) from % membrane integrity of cell monolayers left outside incubator conditions (data represent the mean ± SD, n = 3).

Space limitations prevented the application of the exogenous magnetic field within a temperature- and CO_2_-controlled cell incubator. Controls were conducted to evaluate the effect of exposing PCEC monolayers to room temperature and ambient conditions for 30 minutes and 3 hours (time equivalent to that required for magnetic field treatment). It was observed that cell viability (determined by SYTO 13/GelRed fluorescence imaging) of both groups exposed to ambient conditions for up to 3 hours retained intact membranes (i.e., green-fluorescing cells, Fig 1A). The negative control for a PCEC culture plunged into liquid nitrogen for 1 hour showed damaged membranes and non-viable cells (i.e., red-fluorescing cells, Fig 1A). There was no decrease in relative cell viability of PCECs left outside of CO_2_ incubator conditions after 30 minutes (99.9 ± 0.1%) or after 3 hours (99.9 ± 0.1%) (Fig 1B). Two-sample t-tests on the two groups showed no statistical difference between the relative viabilities of the 30-minute sample and the 3-hour sample (p-value = 0.3). These results suggest that leaving confluent PCEC cultures at ambient conditions for up to 3 hours does not result in a decrease in cell viability. There was also no significant difference observed between the absolute viabilities of the two groups (p-value = 0.9, Fig 1B) suggesting that no cell detachment from the coverslip or monolayer occurs from leaving PCEC monolayer cultures outside of cell culture conditions for up to 3 hours. The absolute viability (Equation 2) captures membrane integrity damage and cell detachment, as well as any increase in total cell count arising from proliferation. Thus, absolute viability values typically have larger variances due to the fact that the number of live cells on the experimental coverslips (numerator in Equation 2) and the total number of cells on the positive control coverslips (denominator in Equation 2, which is an average from multiple samples) are obtained from different monolayer samples of different confluencies. Based on our viability results for control samples, we infer that any changes in cell viability observed from the application of MPs and a rotating magnetic field can be attributed directly to the magnetic treatment without concern of overlapping effects from the environmental conditions.

### Membrane integrity assessment of cell viability of treated PCEC monolayers

Confluent PCEC cultures in cell medium with or without added MPs were subjected to a rotating magnetic field treatment of 5.5 Hz and 0.5 T for 3 hours. Immediately after applying the magnetic field, cultures were stained with SYTO 13/GelRed, and the membrane integrity (cell viability) was analyzed. The treatment groups included bare MPs, conjugated MP-p-IPL, and conjugated MP-p-LPS added to PCEC medium as well as a control group of only cell medium. A total of seven coverslips for the cell medium group and six coverslips for the MP-treated groups were tested.

**Fig 2:**
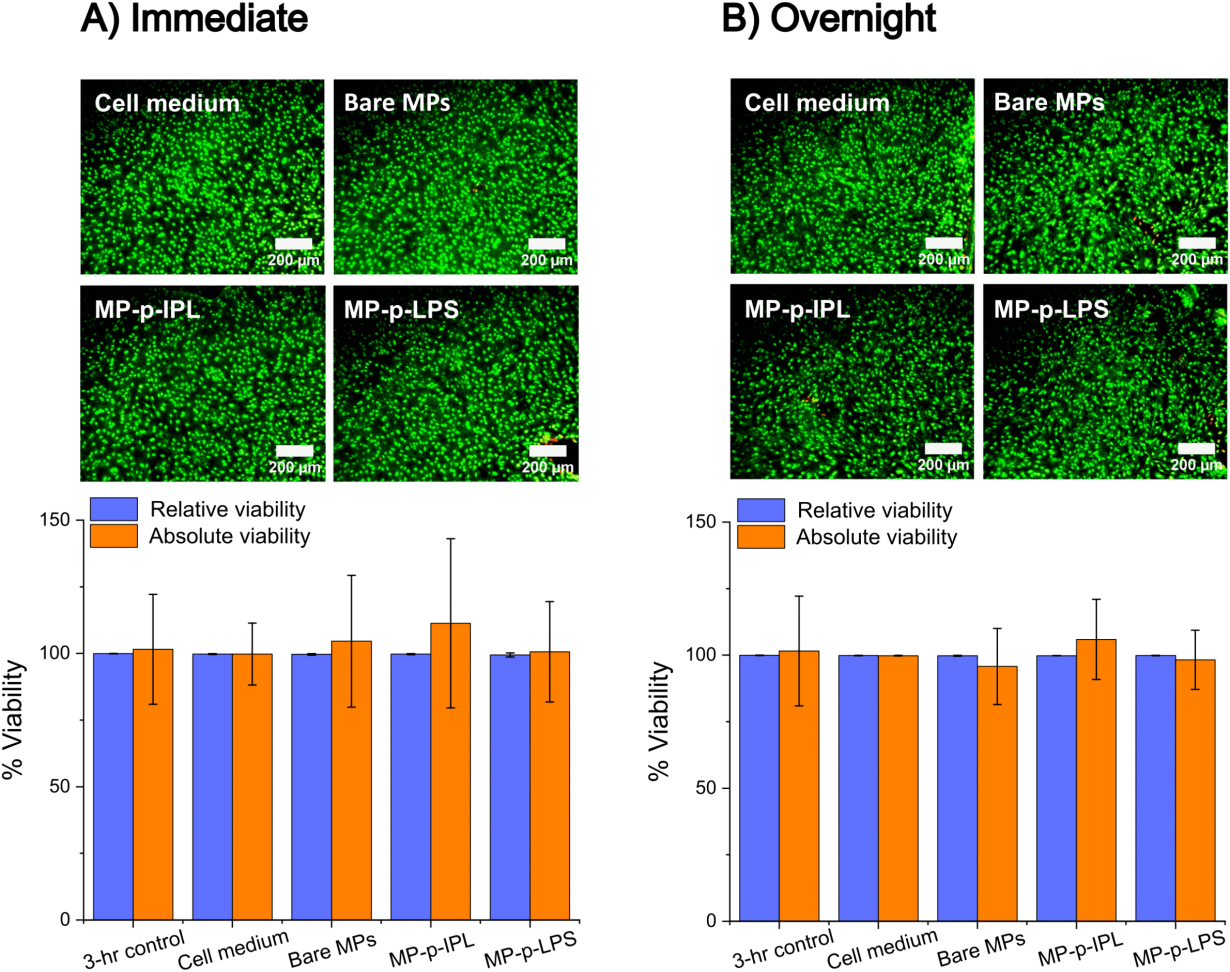
Viability assessments following magnetic field treatment. Porcine corneal endothelial cell monolayers were exposed to magnetic particles (MPs) and a rotating magnetic field and assessed **(A)** immediately after magnetic field application, or **(B)** after an overnight incubation under cell culture conditions following magnetic field application. Representative images (100X magnification) were obtained from porcine corneal endothelial cell monolayers stained with SYTO 13/GelRed. Green fluorescence indicates intact cell membranes and red fluorescence indicates damaged cell membranes. Relative and absolute viabilities were calculated using Equations 1 and 2 in the text, respectively. Data represent the mean ± SD, n ≥ 3; no statistically significant difference was observed between the means (p > 0.05).

All the treated PCEC monolayer cultures stained with SYTO 13/GelRed showed cells with intact membranes (green fluorescence) as shown in Fig 2A. The relative viabilities from the percentage membrane integrity (% MI) assessments of all the groups remained greater than 99% after exposure to the rotating magnetic field (Fig 2A): in cell medium only (99.8 ± 0.2%), in the presence of bare MPs (99.7 ± 0.3%), MP-p-IPL (99.7 ± 0.2%), and MP-p-LPS (99.4 ± 0.8%). One-way ANOVA performed between all the groups (including the 3-hour control not subjected to magnetic field) did not show statistically significant differences between any of the groups (p-value = 0.3).

Absolute viabilities for the treatment groups subjected to both MPs and magnetic field also remained high across the groups after treatment: cell medium only (99.8 ± 11.7%), bare MPs (104.6 ± 24.7%), MP-p-IPL (111.3 ± 31.7%), and MP-p-LPS (100.6 ± 18.8%). One-way ANOVA performed among the absolute viabilities of the treatment groups and the 3-hour no magnetic field control did not show any statistically significant difference (p-value = 0.9).

The lack of statistically significant differences between the relative viabilities of the treated samples and the controls indicates that the application of bare or peptide-conjugated MPs and a rotating magnetic field of 5.5 Hz, 0.5 T for 3 hours did not result in a decrease in the cell viability of PCECs. The lack of statistically significant differences between the absolute viabilities suggests that the magnetic treatment does not cause cells to detach from the monolayer.

To examine any delayed effects on the PCEC cultures, the tests were repeated with four sample groups (three coverslips each): cell medium only, bare MPs, MP-p-IPL, and MP-p-LPS. SYTO 13/GelRed staining and fluorescent imaging assessments were done after overnight incubation under cell culture conditions post-treatment. Fluorescent images of the SYTO 13/GelRed-stained cells show all sample groups with predominantly intact cell membranes (green fluorescence in Fig 2B). The relative viabilities of all the groups assessed after extended incubation were greater than 99% (Fig 2B): cell medium only (99.9 ± 0.1%), bare MPs (99.8 ± 0.2%), MP-p-IPL (99.8 ± 0.1%), and MP-p-LPS (99.8 ± 0.1%). Absolute viabilities for the overnight groups were all greater than 95% (Fig 2B): cell medium only (99.8 ± 0.1%), bare MP (95.8 ± 14.3%), MP-p-IPL (105.9 ± 15.1%), and MP-p-LPS (98.3 ± 11.1%). One-way ANOVA performed between the overnight groups and the control (3-hour without magnetic field treatment) showed no statistically significant difference between the relative viabilities (p = 0.4) and absolute viabilities (p = 0.9) of any of the sample means. Notably, aside from the huge standard deviations in the absolute viabilities as explained above, in both the immediate and overnight samples the absolute viability of the MP-p-IPL groups is greater than 100%. This is not unusual because absolute viability is used as an indication of any cell detachment resulting from magnetic treatment, but ultimately captures any change in cell count (whether it is increasing or decreasing). The immediate test group could simply have had greater confluency than the control group while the overnight test group could have had increased proliferation after overnight incubation. The important finding is that both groups do not indicate damage to the PCEC monolayers following exposure to MPs in a rotating magnetic field. Overall, no significant decrease in cell viability was seen between immediate versus overnight assessment of the magnetic field-treated PCEC cultures, suggesting that there is no acute or prolonged negative effect on the cells following treatment. There is also no evidence of delayed cell detachment from the PCEC monolayer after magnetic treatment, because no significant difference in absolute viability was observed between the immediate and overnight samples after 3 hours of magnetic field exposure and 0.2 µg/µL of MPs.

### Metabolic activity assessment of PCEC monolayers with AlamarBlue

**Fig 3:**
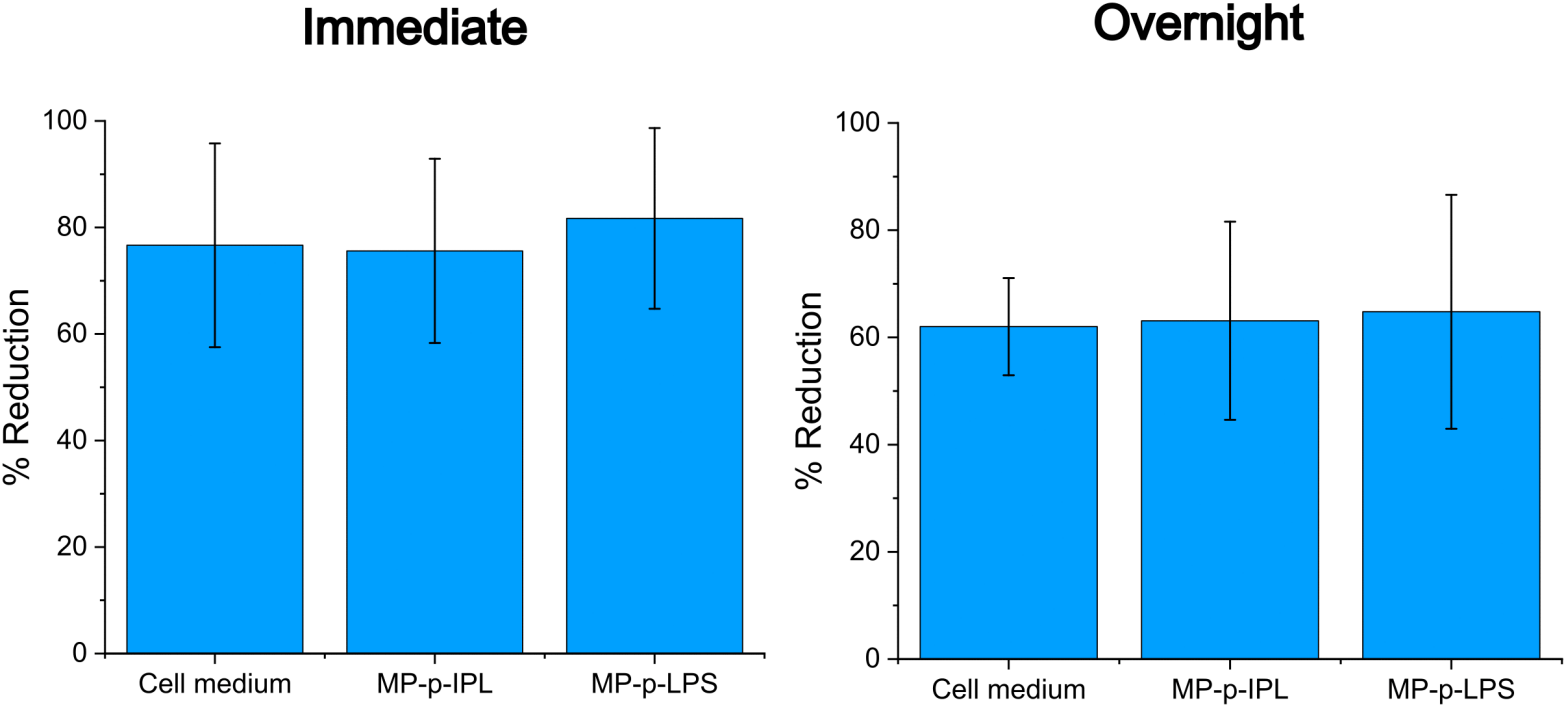
Metabolic activity of porcine corneal endothelial cell cultures following magnetic treatment. Magnetic particles (MP-p-IPL and MP-p-LPS) were added to porcine corneal endothelial cell cultures and subjected to a rotating magnetic field. Plots show the percent reduction of AlamarBlue after 4 hours with assessments performed immediately after magnetic field treatment and after overnight incubation subsequent to magnetic field treatment. Data represent the mean ± SD, n = 3; no statistical difference was observed between the means (p > 0.05).

AlamarBlue reduction was used to assess the metabolic activity of confluent PCEC cultures treated with MPs and a rotating magnetic field. Three sample groups were tested (cell medium only, MP-p-IPL, and MP-p-LPS), and absorbance readings were done either immediately after the 3-hour magnetic treatment or after an overnight period at 5% CO_2_ and 37 °C following magnetic field exposure. AlamarBlue reduction greater than 60% was observed in all the PCEC cultures immediately after magnetic field treatment: cell medium only (76.7 ± 19.1%), MP-p-IPL (75.6 ± 17.3%), MP-p-LPS (81.7 ± 17.0%); and after overnight incubation following magnetic field treatment: cell medium only (62.0 ± 9.1), MP-p-IPL (63.1 ± 18.5%), and MP-p-LPS (64.8 ± 21.8%) (Fig 3). One-way ANOVA performed on all test groups (immediate and overnight samples) showed no statistically significant difference between the group means (p-value = 0.9).

The metabolic activity of the PCEC cultures was confirmed after treatment with MPs and a rotating magnetic field immediately after a 3-hour exposure and after overnight incubation following magnetic treatment. This suggests that the cell viability and metabolic function of the PCEC cultures are maintained after magnetic treatment.

### Immunocytochemical staining of ZO-1

**Fig 4:**
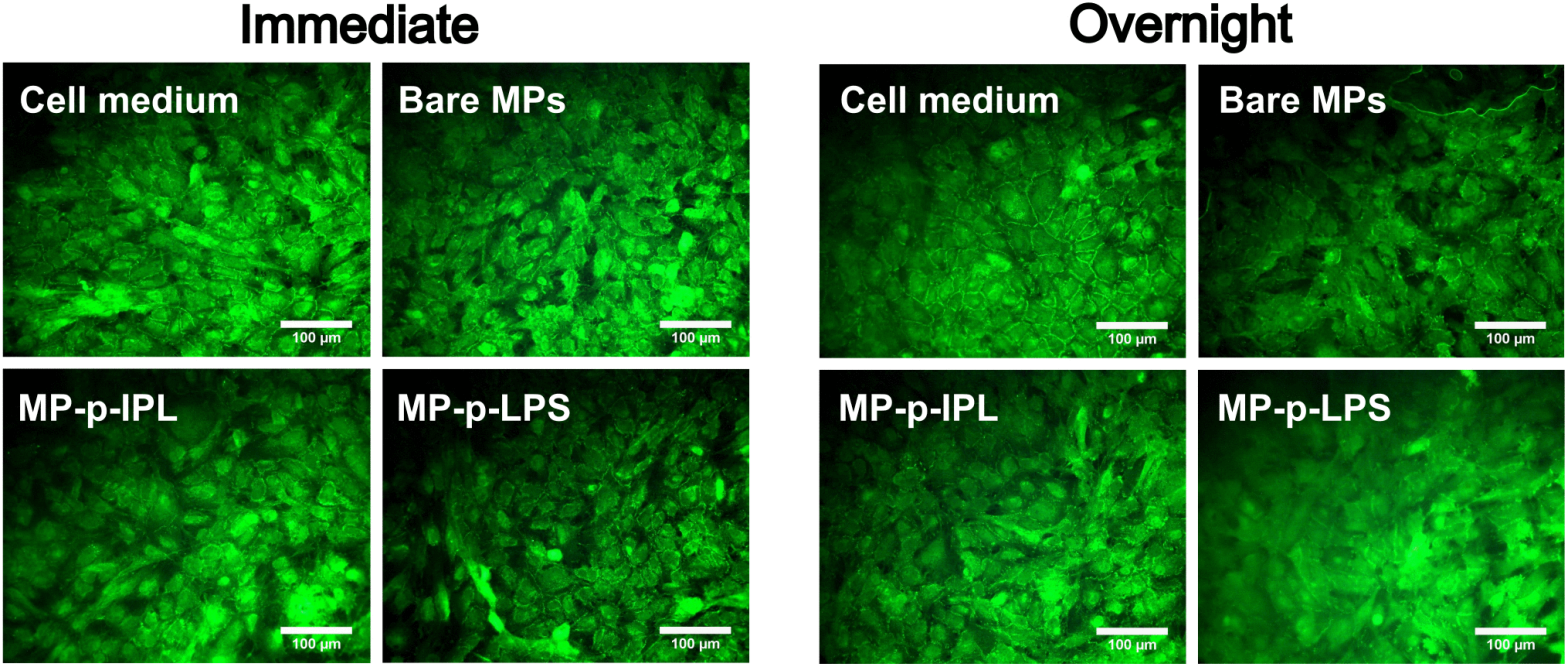
ZO-1 tight junction expression in porcine corneal endothelial cell monolayers after magnetic treatment. Porcine corneal endothelial cell cultures were exposed to magnetic particles (MPs) and a rotating magnetic field. Fluorescence images (250X magnification) of PCEC monolayers immunocytochemically stained with ZO-1 and imaged immediately after magnetic field treatment and after an overnight incubation under cell culture conditions post-treatment.

Immunocytochemical staining for the tight junction protein ZO-1 was done to confirm the stability of the monolayer structure of the PCEC cultures after exposure to MPs and a rotating magnetic field. Sample groups with medium only, bare MPs, MP-p-IPL, or MP-p-LPS were assessed immediately after a 3-hour rotating magnetic field application or after an overnight incubation under cell culture conditions post-treatment. Fluorescence images of the ZO-1 immunocytochemical staining in Fig 4 show the ZO-1 tight junction protein present at the periphery of cells in all the PCEC monolayers after exposure to MPs and a rotating magnetic field. Although ZO-1 is usually localized along the borders of adjacent cells in an endothelial monolayer, it has been reported to also manifest in subcellular structures such as the nucleus and the cytoplasm [39,40]. The presence of ZO-1 in both control and treated groups indicates that the monolayer structure of the PCEC cultures is still intact after magnetic field treatment, suggesting that there is no immediate or delayed damage to the structure of PCEC monolayer cultures following MP (0.2 µg/µL) and rotating magnetic field (5.5 Hz and 0.5 T) exposure.

## Discussion

Some iron oxide nanoparticles have been shown to interfere with fluorometric or absorbance-based assays [41,42]. Herein, no interference induced by the MPs on these assays is expected because we remove the MP solution from the cell monolayers prior to conducting any assessment, and we use large particles (i.e., 1-µm) that do not show any fluorescence [43,44].

The results of cell viability, metabolic activity, and immunocytochemical assessments showed no significant decrease in PCEC viability and metabolic activity and no damage to the monolayer structure following a 3-hour exposure to 0.2 µg/µL of MPs and a 5.5 Hz, 0.5 T rotating magnetic field. In agreement with previous studies that have used stationary magnetic fields to direct magnetized cultured cells toward the cornea without detecting endothelial cell injury [23,27–29], our work shows that exposure of MPs to PCEC cultures for 3 hours in a relatively strong rotating magnetic field did not decrease cell viability. Notably, the exposure time of MPs to the cell cultures was much shorter in our study than in the cell delivery studies (> 24 hours [27]), suggesting that the PCEC cultures may remain viable when exposed to MPs for durations longer than the 3 hours employed here. The viability assessments done in this study were also performed after at most an overnight incubation time as opposed to the longer observation times performed in the cell delivery studies for up to 7 days *in vitro* [28]. The absence of significant differences suggests that exposure to MPs and a rotating magnetic field did not induce cell necrosis which would have been observed immediately. Moreover, the lack of any gradual decrease in viability even after overnight incubation suggests that the cells were also not undergoing apoptosis. The size of MPs used in the cell delivery studies were also much smaller (50 nm) than our 1-μm particles. One set of cell viability experiments on PCEC monolayers was performed using bare 50-nm MPs (S1 File), in which no decrease in cell viability was observed, agreeing with the cell delivery studies. There is no further evidence, however, that 1-μm particles are safe with corneal endothelial cells for longer than 3 hours. Size-dependent toxicity of MPs in rodent eyes was observed where a significant decrease in corneal endothelial cell numbers was seen at 1 week and 5 months after injection of rat eyes with 4-µm sized MPs [45]. No significant difference was observed in the eyes injected with 50-nm MPs compared to PBS over the course of 5 months [45]. This suggests a potential size threshold, above which negative effects are imposed on corneal endothelial cells upon injection of MPs into the anterior chamber for long time durations (more than 1 week). Consequently, unless there is a physical removal of the 1-μm peptide-conjugated MPs following injection and magnetic field application in the anterior chamber, 50-nm MPs are likely a safer option for the magnetic removal of pseudoexfoliation material from lens capsules *in vivo*.

Another important difference between our study and the cell delivery studies is the application of the external magnetic field. The delivery studies employed a static magnetic field, typically with a permanent neodymium magnet for various times (3–24 hours), whereas we used a dynamic rotating magnetic field. The cell delivery studies examined the physical movement of the corneal endothelial cells whereas our study determined whether moving MPs in close proximity to the cell monolayers cause any deleterious effects. It is assumed that the MPs are not taken up by PCECs during the 3-hour duration of our experiments; however, Moysidis et al. [28] observed intracellular MPs in human corneal endothelial cell cultures after a 3-hour incubation with MPs. It becomes concerning if any PCECs that endocytosed MPs could be detached from the monolayer upon exposure to magnetic forces applied by a rotating magnetic field. The cultured corneal endothelial cells injected into anterior chambers in the aforementioned studies were shown to create a monolayer on the posterior corneal surface, showing tight junction markers. The ability of the initially free-floating cells to settle and create a functional monolayer in the anterior chamber suggests that even if cells were to detach from a monolayer following application of MPs and a rotating magnetic field, as long as the cells are viable and in close proximity to the posterior corneal surface, they can reattach and reintegrate themselves. This is likely as the rotating magnetic field pulls laterally, meaning any cells disrupted by the magnetic field would still be in contact with the monolayer. However, the overall ability of corneal endothelial cells to endocytose moving particles is unlikely because the application of the rotating magnetic field occurs immediately after the addition of MPs to the PCEC cultures.

The results of this study, supporting the safe therapeutic potential of MPs and a low-frequency rotating magnetic field on PCEC monolayers, still requires investigation into the safety of this therapy *in vivo* and any effects it may have on the ocular system as a whole prior to clinical translation. Future investigation into the effects of different parameters such as size and concentration of MPs, frequency and strength of the applied magnetic field, and duration of magnetic exposure on corneal endothelial monolayers will also help facilitate the development of a safe clinical therapy not just for pseudoexfoliation syndrome, but potentially other ocular diseases as well.

## Conclusions

The results of membrane integrity fluorescence imaging with SYTO 13 and GelRed staining showed no decrease in cell viability immediately after treatment of PCEC monolayers with a rotating magnetic field, with or without MPs. No decrease in cell viability was observed after overnight incubation of PCEC monolayers after magnetic field exposure. The cells also exhibited metabolic activity by AlamarBlue reduction following MP/magnetic-field application both immediately and after extended incubation. The ZO-1 tight junction protein expression was shown by immunocytochemical staining in all samples, showing that the cell monolayer was not structurally compromised after treatment. Taken together, our results suggest that the application of 1-µm peptide-conjugated MPs (at 0.2 µg/µL) coupled with a 5.5 Hz, 0.5 T rotating magnetic field for up to 3 hours does not cause negative effects on PCECs through membrane integrity, metabolic activity, and tight junction structural assessments. Our findings using PCEC monolayers, a model closely resembling human corneal endothelium, support the promising potential of this magnetic treatment in the anterior chamber of the eye to safely remove pseudoexfoliation materials from the lens capsule.

## Supporting information

S1 File Supporting information. Porcine corneal endothelial cell viability following exposure to 50-nm magnetic particles and a rotating magnetic field.
(DOCX)

S2 File Raw data. Membrane integrity and AlamarBlue absorbance data.
(XLSX)
