# Supplementary material for: Stability of corneal endothelial monolayers in the presence of magnetic particles in a rotating magnetic field
Source: PLoS One. 2026 May 6;21(5):e0348603. doi: 10.1371/journal.pone.0348603 (PMC13148704; doi:10.1371/journal.pone.0348603)
Supplement: S1 File — Porcine corneal endothelial cell viability following exposure to 50-nm magnetic particles and a rotating magnetic field. (DOCX) [file pone.0348603.s001.docx]

Stability of corneal endothelial monolayers in the presence of magnetic particles in a rotating magnetic field.

Jessica Chin ^1^, Leah A. Marquez-Curtis ^2,3^, Mehdi Ghaffari Sharaf ^2^, Janet A. W. Elliott ^2,3*^, and Larry D. Unsworth ^1,2*^

^1^ Department of Biomedical Engineering, University of Alberta, Edmonton, Alberta, Canada

^2^ Department of Chemical and Materials Engineering, University of Alberta, Edmonton, Alberta, Canada

^3^ Department of Laboratory Medicine and Pathology, University of Alberta, Edmonton, Alberta, Canada

* Corresponding Authors

E-mails: janet.elliott@ualberta.ca (JAWE), larry.unsworth@ualberta.ca (LDU)

**Supporting Information**

An immediate membrane integrity assessment was also performed in triplicate on porcine corneal endothelial cell (PCEC) cultures after bare 50-nm magnetic particles (MPs; Nanocs Inc., New York, USA) were added at a concentration of 0.2 μg/μL and rotating magnetic field applied. A representative image of the SYTO 13/GelRed stained culture is shown in S1 Fig. No statistically significant difference was observed in the relative viability (99.4 ± 0.34%) and absolute viability (96.0 ± 22.6%) between the 50-nm MP group and the other test groups (relative viability: p-value = 0.3; absolute viability: p-value = 0.9). This shows that treatment with smaller 50-nm MPs (0.2 μg/μL) coupled with a rotating magnetic field of 5.5 Hz and 0.5 T for up to 3 hours also does not decrease PCEC cell viability.


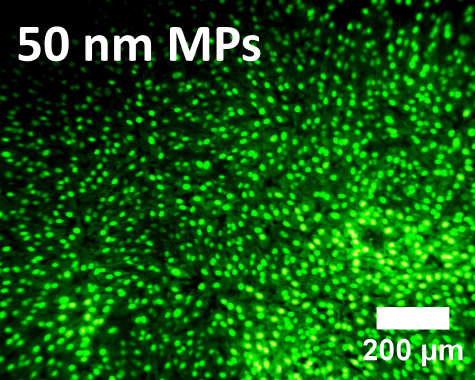


**S1 Fig. Intact cell membranes following exposure to 50-nm magnetic particles and rotating magnetic field.** Representative image (100X magnification) of SYTO 13/GelRed-stained porcine corneal endothelial cell monolayer treated with 50-nm magnetic particles (MPs; 0.2 μg/μL) and a rotating magnetic field of 5.5 Hz and 0.5 T for up to 3 hours.
